# Supplementary material for: COZOID: contact zone identifier for visual analysis of protein-protein interactions
Source: BMC Bioinformatics. 2018 Apr 6;19:125. doi: 10.1186/s12859-018-2113-6 (PMC5889581; doi:10.1186/s12859-018-2113-6)
Supplement: Supplementary file 2 — Software build. Executable binary file of the software tool. (ZIP 118,784 kb) [file 12859_2018_2113_MOESM2_ESM.zip › cozoid/platform/docs/testng-6.5.1-javadoc.zip!/org/testng/ITestClassFinder.html]

ITestClassFinder (TestNG)


---


|  |  |  |
| --- | --- | --- |
| **Overview** \| **Package** \| **Class** \| **Tree** \| **Deprecated** \| **Index** \| **Help** | |  |
| **PREV CLASS**   **NEXT CLASS** | **FRAMES**    **NO FRAMES**     **All Classes** |  |
| SUMMARY: NESTED \| FIELD \| CONSTR \| METHOD | DETAIL: FIELD \| CONSTR \| METHOD |  |


---


## org.testng Interface ITestClassFinder

---

``` public interface ITestClassFinder ```

This class is used by TestNG to locate the test classes.

**Author:**
:   Cedric Beust

---

| **Method Summary** | |
| --- | --- |
| `IClass[]` | `findTestClasses()` |
| `IClass` | `getIClass(java.lang.Class cls)`             Return the IClass for a given class |

**Method Detail**

### findTestClasses

```
IClass[] findTestClasses()
```

:   **Returns:**: An array of all the classes that contain test methods. This method usually returns an array of one class, which is the class on which TestNG is running, except in the following cases. - TestNG: the class contains an @Factory method - JUnit: the class contains a suite() method

---


### getIClass

```
IClass getIClass(java.lang.Class cls)
```

:   Return the IClass for a given class


---


|  |  |  |
| --- | --- | --- |
| **Overview** \| **Package** \| **Class** \| **Tree** \| **Deprecated** \| **Index** \| **Help** | |  |
| **PREV CLASS**   **NEXT CLASS** | **FRAMES**    **NO FRAMES**     **All Classes** |  |
| SUMMARY: NESTED \| FIELD \| CONSTR \| METHOD | DETAIL: FIELD \| CONSTR \| METHOD |  |


---
